# Transversus abdominis-plane block versus local anesthetic wound infiltration in lower abdominal surgery: a systematic review and meta-analysis of randomized controlled trials

**DOI:** 10.1186/1471-2253-14-121

**Published:** 2014-12-15

**Authors:** Nanze Yu, Xiao Long, Jorge R Lujan-Hernandez, Julien Succar, Xin Xin, Xiaojun Wang

**Affiliations:** Division of Plastic Surgery, Peking Union Medical College Hospital, Peking Union Medical College, Chinese Academy of Medical Science, No.1 Shuaifuyuan, Wangfujing, Dongcheng District, Beijing, 100730 China; Division of Plastic Surgery, Brigham and Women’s Hospital and Harvard Medical School, Boston, MA USA; Department of Surgery, University of Kentucky, Lexington, KY USA; Department of Anesthesiology, Peking Union Medical College Hospital, Peking Union Medical College, Chinese Academy of Medical Science, Beijing, China

**Keywords:** Transversus abdominis plane block, Local anesthetic infiltration, Postoperative analgesia, Meta-analysis

## Abstract

**Background:**

Postoperative pain management is of great importance in perioperative anesthetic care. Transversus abdominis plane (TAP) block has been described as an effective technique to reduce postoperative pain and morphine consumption after open lower abdominal operations. Meanwhile, local anesthetic infiltration (LAI) is also commonly used as a traditional method. However, the effectiveness of these two methods has not been compared before.

**Methods:**

A meta-analysis of all relevant randomized controlled trials (RCTs) was conducted to compare the efficacy of single shot TAP block with that of single shot LAI for postoperative analgesia in adults. Major medical databases and trial registries were searched for published and unpublished RCTs. The endpoints include postoperative visual analog scale (VAS) pain score, morphine requirement, and rate of postoperative nausea and vomiting (PONV). For continuous data, weighted mean differences (WMDs) were formulated; for dichotomous data, risk ratios (RR) were calculated. Results were derived using a random-/fixed-effects model with 95% confidence interval (CI).

**Results:**

Four RCTs, encompassing 96 TAP-block and 100 LAI patients, were included in the final analysis. Patients in the TAP-block group had lower VAS pain scores 24 hours postoperatively compared with the LAI group, both at rest (WMD [95% CI] = -0.67 [p < 0.01] and with movement (WMD = -0.89, p < 0.01). There were no significant between-group differences in 24-hour postoperative morphine requirements, the rates if PONV or VAS pain scores at 2 and 4 h postoperatively.

**Conclusion:**

TAP block and LAI provide comparable short-term postoperative analgesia, but TAP block has better long-lasting effect.

**Electronic supplementary material:**

The online version of this article (doi:10.1186/1471-2253-14-121) contains supplementary material, which is available to authorized users.

## Background

Acute postoperative pain is a common problem encountered not only by pain specialists, but also by all medical professionals in everyday practice [1]. Pain management is an important aspect of perioperative anesthetic care, while whether acute postoperative pain control affects surgical outcomes remains controversial [2]. There is general agreement that it is a major reason for primary care consultation and a cause of prolonged hospital stays and patient dissatisfaction [3].

Transversus abdominis plane (TAP) block, first described by Kuppuvelumani et al. in 1993 [4] and formally documented by Rafi in 2001 [5], is used for the management of surgical abdominal pain by injecting local anesthesia into the plane between the internal oblique and transversus abdominis muscle [5, 6]. TAP-block technique has been shown to be a safe and effective postoperative adjunct analgesia method in a variety of general [7, 8], gynecological [9–11], urological [12], plastic [13, 14], and pediatric [15, 16] surgeries, and it is suggested as part of the multimodal anesthetic approach to enhance recovery after lower abdominal surgeries [17].

Single shot local anesthetic infiltration (LAI) is also a commonly used method for reducing postoperative pain [18, 19]. Pain relief can be obtained by single injection of local anesthesia into skin and subcutaneous tissue layer at surgical incision sites, which could lower the pain scores until 24 hours postoperatively [20]. There have been a number of randomized controlled trials (RCTs) comparing the efficacy of TAP block to that of LAI, but the results are inconsistent. Thus, we conducted a meta-analysis of all RCTs in this area to determine whether TAP block is more efficacious during the postoperative period of lower abdominal surgery in adults.

## Methods

### Study identification

A comprehensive literature search was performed using the following search terms: *transvers abdominis plane block* or *transverse abdominis plane block* or *TAP block*. No limitations with respect to sex, human or animal studies, language, or publication year were applied. The search was performed independently by two authors (N. Y. and X. L.) according to the validated methods of the PRISMA statement [21]. The databases, searched prior to 1 January 2014, were PubMed (1966–2014), MEDLINE® (1966–2014), EMBASE (1974–2014), Cochrane Central Register of Controlled Trials (1996–2014), and Cumulative Index to Nursing and Allied Health Literature (CINAHL) (1983–2014). Unpublished trials and conference proceedings were searched with the System for Information on Grey Literature in Europe, the National Research Register (UK), and trial registries. Papers were also searched among those quoted as references in the retrieved studies to ensure the relevant studies were included and the language was limited to English only.

### Study selection

Articles were included if they met the following criteria:

- Study design: RCT
- Population: Human adults (18 years old and older) who underwent lower abdominal surgery
- Intervention: single-shot TAP block compared with single-shot LAI
- Outcomes:
  1. Efficacy (postoperative pain at rest/with movement);
  2. Postoperative morphine requirement;
  3. Postoperative nausea and vomiting (PONV)

**Figure 1:**
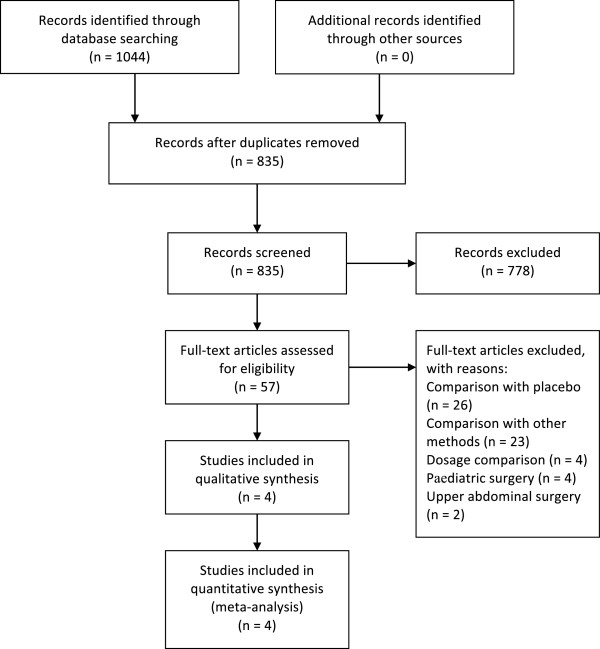
**Study flow diagram.**

Exclusion criteria were as follows:

- Non-randomized studies
- TAP block compared with placebo or other methods, or TAP-block dosage comparison
- Studies involving preperitoneal injection
- Pediatric surgery
- Letters, case reports, reviews, comments, and editorials
- Languages other than English
- Animal studiesStudies were considered for inclusion independently by two authors (N. Y. and X. L.) and any disagreements were resolved by consensus after consultation with a senior author (X.W.) (Figure 1). Each article was critically reviewed for eligibility in our analysis.

### Data extraction

A table was designed to include the most relevant variables common to the papers assessed (Table 1). The outcomes of interest were postoperative VAS pain scores, morphine requirements, and rates of PONV. Two authors (N. Y. and X. L.) independently extracted data from eligible articles and any discrepancy was resolved prior to final analysis. Missing information and inadequate data were sought from corresponding authors through email correspondence over a period of 2 months, and if there was no response, the data were considered incomplete.

**Table 1:** **Characteristics of included studies**

Author	Operation performed	Group	N	Anesthetic used	VAS pain score at rest			VAS pain score on movement			Morphine(mg)	PONV(%)
					2 h	4 h	24 h	2 h	4 h	24 h	24 h	24 h
Atim 2011	Hysterectomy	TAP	18	0.25% bupivacaine, 20 ml each side	2.5 ± 1.7	2.0 ± 2.1	0.3 ± 0.4	2.8 ± 1.7	2.4 ± 1.7	0.3 ± 0.4		
		LAI	19	0.25% bupivacaine, 20 ml total	3.7 ± 2.2	2.7 ± 1.3	1.0 ± 0.9	4.1 ± 2.6	2.8 ± 1.3	1.2 ± 0.9		
Sivapurapu 2013	Gynecological surgery	TAP	26	0.25% bupivacaine 0.6 ml/kg, total	4.0 ± 1.3	3.6 ± 0.8	3.5 ± 0.7				22.15 ± 4.14	4
		LAI	26	0.25% bupivacaine 0.6 ml/kg, total	6.7 ± 1.4	5.1 ± 0.7	4.7 ± 0.6				29.15 ± 3.93	23
Petersen 2013	Inguinal hernia repair	TAP	29	0.75% ropivacaine, 25 ml each side	2.8 ± 1.5	3.1 ± 1.8	1.7 ± 1.3	4.3 ± 1.8	4.6 ± 1.9	3.7 ± 1.8	6.6 ± 11.5	17
		LAI	30	0.375% ropivacaine, 40 ml total	0.8 ± 1.1	1.2 ± 1.3	2.0 ± 1.5	1.3 ± 1.6	2.0 ± 1.8	4.5 ± 2.3	2.1 ± 5.1	20
Skjelsager 2013	Open radical prostatectomy	TAP	23	0.75% ropivacaine, 40 ml total	1.8 ± 1.6	1.2 ± 1.3	0.6 ± 1.0	2.9 ± 2.3	2.8 ± 2.2	1.6 ± 1.4		30
		LAI	25	0.75% ropivacaine, 40 ml total	2.0 ± 1.5	1.6 ± 1.2	0.9 ± 0.8	3.8 ± 2.1	3.3 ± 1.8	2.5 ± 1.9		25

### Statistical analysis

Data analysis was performed with Revman 5.2 (The Cochrane Collaboration, Copenhagen, Denmark) and Stata Statistical Software, Release 9 (StataCorp. LP, College Station, TX, USA). For continuous data, Hedges’ g statistic was used to calculate weighted mean difference (WMD) and 95% confidence interval (CI). Inverse variance was used for continuous outcome variables. Binary data was summarized as risk ratios (RR) and 95% CI. The heterogeneity of the estimators was tested using I^2^. When I^2^ was lower than 50%, the studies were considered to have acceptable heterogeneity and the fixed-effect model with Mantel-Haenszel method was then used; otherwise, a random-effects model with the DerSimonian and Laird (DL) method was adopted. Forest plots were constructed. The pooled effect and its 95% CI were represented by a diamond that did not cross the vertical line of no effect (standard error of mean difference [SMD] = 0), and P < 0.05 was considered to be statistically significant.

Potential publication bias was investigated with funnel plot of the SMD against the mean difference of the study, which was used as the main graphical method. To supplement the funnel-plot approach, Egger linear regression was used to test for publication bias using quantitative analysis.

## Results

The database search produced 1044 studies, of which four were eligible for analysis after applying exclusion criteria [8–10, 12]. The quality of the included studies was assessed with a combined-criteria score system, as set out by Jadad et al. [22] and Chalmers et al. [23] (Table 2). Totally there were 96 patients of the TAP-block group and 100 of the LAI group included in this study. We deleted a single study from the overall pooled analysis each time to check sensitivity; the result did not significantly affect the overall estimate.

**Table 2:** **Modified quality score for randomized trials**†

Quality variables	Atim 2011	Sivapurapu 2013	Petersen 2013	Skjelsager 2013
Was study described as randomized, ie, used words such as “randomly”, “random”, and “randomization”? [0,1]	1	1	1	1
Was randomization described, and appropriate? [-1,0,1]	1	1	1	1
Was study described as double-blinded? [0,1]	1	0	1	1
Was method of blinding appropriate? [-1,0,1]	1	1	1	1
Was a description of withdrawals and dropouts included? [0,1]	1	0	1	1
Inclusion criteria	1	1	1	1
Exclusion criteria	1	1	1	1
Study period given	0	0	1	1
Appropriate statistical analysis	1	1	1	1
Hard end points	1	1	1	1
Sample size calculation	1	1	1	1
Baseline characteristics comparable	1	1	1	1
Any postoperative data missing?	1	1	1	1
Allocation concealment	1	0	1	1
Analysis by intention to treat	0	0	0	0
Score^‡^	13	10	14	14

^†^Score maximum = 15. Poor = -1 to 5, Fair = 6 to 10, Good = 11 to 15.

^‡^-1 = inappropriate or inaccurate, 0 = not given/inadequate information, 1 = described and accurate.

### VAS scores at 2 hours postoperation

**Figure 2:**
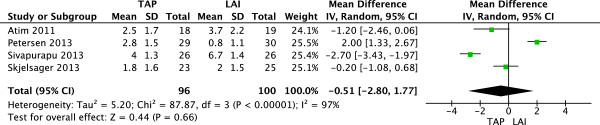
**Random-effects meta-analysis of mean VAS pain score at rest 2 h following surgery with TAP block and LAI.** LAI, local anesthetic infiltration; TAP, transversus abdominis plane; VAS, visual analog scale.

Four studies [8–10, 12] (196 patients; 96 TAP block, 100 LAI) reported VAS scores at rest 2 h after surgery. There was significant heterogeneity among the studies (I^2^ = 97%, P < 0.00001). A random-effects model was used, there was no significant difference in mean VAS pain score 2 h postoperatively between patients who received LAI and those with TAP block (Figure 2).

**Figure 3:**
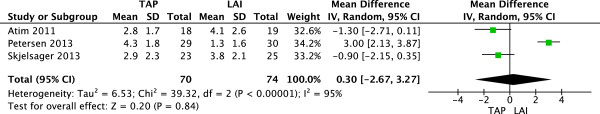
**Random-effects meta-analysis of mean VAS pain score with movement 2 hours following surgery with TAP block and LAI.** LAI, local anesthetic infiltration; TAP, transversus abdominis plane; VAS, visual analog scale.

Three studies [8, 9, 12] (144 patients; 70 TAP block, 74 LAI) reported VAS scores with movement 2 h after surgery. There was significant heterogeneity among the studies (I^2^ = 95%, P < 0.00001). A random-effects model was used, there was no significant difference in mean VAS pain score 2 h postoperatively between patients who received LAI and those with TAP block (Figure 3).

### VAS scores at 4 hours postoperation

**Figure 4:**
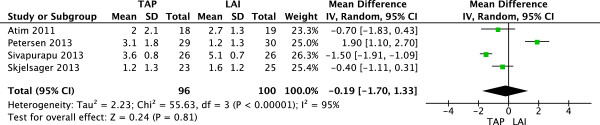
**Random-effects meta-analysis of mean VAS pain score at rest 4 hours following surgery with TAP block and LAI.** LAI, local anesthetic infiltration; TAP, transversus abdominis plane; VAS, visual analog scale.

Four studies [8–10, 12] (196 patients; 96 TAP block, 100 LAI) reported VAS scores at rest 4 h after surgery. There was significant heterogeneity among the studies (I^2^ = 95%, P < 0.00001). A random-effects model was used, and there was no significant difference in mean VAS pain score 4 h postoperatively between patients who received LAI and those with TAP block (Figure 4).

**Figure 5:**
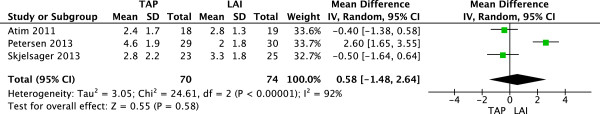
**Random-effects meta-analysis of mean VAS pain score with movement 4 hours following surgery with TAP block and LAI.** LAI, local anesthetic infiltration; TAP, transversus abdominis plane; VAS, visual analog scale.

Three studies [8, 9, 12] (144 patients; 70 TAP block, 74 local infiltration) reported VAS scores with movement at 4 h after surgery. There was significant heterogeneity among the studies (I^2^ = 92%, P < 0.00001). A random-effects model was used, and there was no significant difference in mean VAS pain score at 4 h between patients who had received LAI and those who had received TAP block (Figure 5).

### VAS scores after 24 hours postoperation

**Figure 6:**
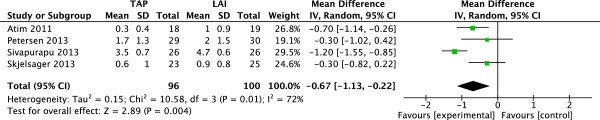
**Random-effects meta-analysis of mean VAS pain score at rest 24 h following surgery with TAP block and LAI.** LAI, local anesthetic infiltration; TAP, transversus abdominis plane; VAS, visual analog scale.

Four studies [8–10, 12] (196 patients; 96 TAP block, 100 LAI) reported VAS scores at rest 24 h after surgery. There was significant heterogeneity among the studies (I^2^ = 72%, P = 0.01). A random-effects model was used, and there was significant reduction in mean VAS pain score at 24 h postoperation in patients received TAP block compared with those who had LAI (Figure 6).

**Figure 7:**
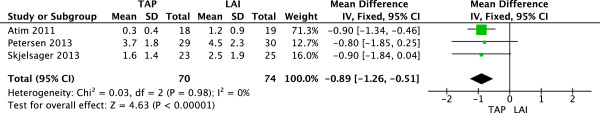
**Fixed-effects meta-analysis of mean VAS pain score on movement 24 h following surgery with TAP block and LAI.** LAI, local anesthetic infiltration; TAP, transversus abdominis plane; VAS, visual analog scale.

Three studies [8, 9, 12] (144 patients; 70 TAP block, 74 LAI) reported VAS scores with movement 24 h after surgery. There was no significant heterogeneity among the studies (I^2^ = 0%, P = 0.98). A fixed-effects model was used, and there was significant reduction in mean VAS pain score at 24 h postoperation in patients received TAP block compared with those who had LAI (Figure 7).

### Morphine requirements after 24 hours postoperation

**Figure 8:**
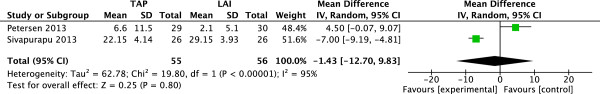
**Random-effects meta-analysis of mean morphine requirement (mg) 24 h following surgery with TAP block and LAI.** LAI, local anesthetic infiltration; TAP, transversus abdominis plane; VAS, visual analog scale.

Two studies [8, 10] (114 patients; 55 TAP block, 56 LAI) reported morphine requirements (mg) 24 h after surgery. There was significant heterogeneity among the studies (I^2^ = 95%, P < 0.00001). A random-effects model was used, and there was no significant difference in mean morphine requirements at 24 h between patients received TAP block and those with LAI (Figure 8).

### PONV rate at 24 hours

**Figure 9:**
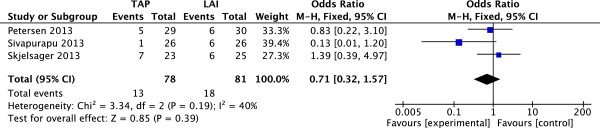
**Fixed-effects meta-analysis of rate of PONV 24 h following surgery with TAP block and LAI.** LAI, local anesthetic infiltration; PONV, postoperative nausea and vomiting; TAP: transversus abdominis plane.

Three studies [8, 10, 12] (159 patients; 78 TAP block, 81 LAI) reported rate of PONV at 24 h after surgery. There was no significant heterogeneity among the studies (I^2^ = 40%, P = 0.19). A fixed-effects model was used, and there was no significant difference in mean rate of PONV at 24 h between the TAP group and LAI group (Figure 9).

### Risk of bias and publication bias

Overall, the included RCTs were of moderate to high quality, as shown in Additional file 1: Figures S1-S10. Funnel plots are also shown in the Additional file 1: Figures S1-S10. However, Egger’s linear regression revealed no evidence of publication bias in this meta-analysis (P = 0.625).

## Discussion

Postoperative pain management is one of the main concerns of both the surgeons and theirs patients. Multiple methods have been put into use to achieve the ideal of pain-free recovery such as LAI [20], epidural analgesia [24], peripheral nerve block [25], and intravenous patient-controlled analgesia [26]. TAP block, which can be easily performed under ultrasound guidance [27] or using a landmark-based approach [28, 29], is becoming more and more commonly applied in lower abdominal surgeries to decrease postoperative pain. TAP block is currently described as an effective technique for reducing postoperative pain and morphine consumption after lower abdominal surgery [17, 30, 31]. Meanwhile, LAI is a convenient postoperative analgesia method, which have been widely performed. Thus it would be helpful to figure out which technique will be more effective in postoperative pain control. To the best of our knowledge, this is the first meta-analysis that has been performed on this topic.

Postoperative pain alleviation is our primary outcome. VAS pain score, considered the gold standard of pain quantification [32], was used to evaluate postoperative pain severity on a scale of 1 to 10 in all the included studies, both at rest and with movement. Our review found that there was a significantly lower pain score in the TAP group at 24 hours postoperatively. However, no significant difference was detected at any other time point, which suggests that TAP block is effective for relatively long-lasting analgesia. LAI is limited to a short period of pain control, and the effect reaches the peak at 1 h postoperatively; then the effect decreases to the minimum by 8 h, and is negligible at 16 h [33]. While TAP block demonstrates its advantage gradually over time. The results of this study are consistent with the observations reported by Ortiz and other researchers [34] that the efficacy of TAP is of longer duration than that of LAI. However, it should be noted that the improvement in pain scores differed by less than one point between TAP AND LAI groups, both at rest and with movement 24 hours after surgery. It suggests that even though it is statistically significant, the two methods may not be clinically significant as it may be challenging for a patient to tell the difference between a VAS score of 2 and 3. In addition, in patients with VAS scores less than 3.4, a mean change of 1.3 could be considered as clinically significant [35]. Therefore it is still uncertain that TAP provides better long-lasting clinical results.

Reducing of postoperative morphine requirement and associated side effects are also desirable, as these are considered detrimental to patient’s recovery [36]. TAP block is effective in reducing both 24 h postoperative morphine requirements and PONV compared with placebo [17]; meanwhile other studies have shown that LAI also decreases postoperative opiate requirements [37] and nausea and vomiting [17] compared with placebo. Data analyzed in this study demonstrated that TAP block did not significantly reduce morphine requirements or PONV compared with LAI 24 h after surgery, suggesting that both methods have good postoperative analgesic effect.

No complication was reported in the included studies. However, TAP block has complications including block failure, vascular injury, abdominal viscera and nerve injuries [38]. Ultrasound-guided TAP block, which allows more accurate visualization of the needle, TAP plane, and injection spot, is considered to be safer clinically.

Our meta-analysis is limited by the small sample size of included studies and by the significant heterogeneity of the outcomes. There were only four studies, totally 196 patients included. During data collection, all the authors of the studies included were emailed three times over a period of 2 months to require the raw data for further assessment and reducing bias, but few of them responded. The heterogeneity is related with different types of surgeries performed and the type and dose of analgesia (which will affect the efficacy and duration of block). Further limitations include differences in block technique and timing of administration.

Our review findings raise another important clinical question regarding time and cost. LAI, performed under direct visualization, is simple and quick; ultrasound-guided TAP block is operator-dependent and time-consuming; thus, future research are required to demonstrate the time requirements and cost efficiency of these two methods.

## Conclusions

TAP block is comparable to LAI for short-term analgesia; it could also provide better long-lasting analgesia especially at 24 h after surgery. Current evidence is insufficient to conclude that TAP block could reduce the requirements for postoperative morphine and associated side effects as compared to LAI. Further RCTs should be performed to figure out the different benefit of these two methods.

## Electronic supplementary material

Additional file 1: Figure S1: Risk of bias graph: review authors’ judgments about each risk of bias item presented as percentages across all included studies. **Figure S2.** Risk of bias summary: review authors’ judgments about each risk of bias item for each included study. **Figure S3.** Funnel plot of VAS score in 2 hours at rest between TAP and LAI groups. **Figure S4.** Funnel plot of VAS score in 2 hours on movement between TAP and LAI groups. **Figure S5.** Funnel plot of VAS score in 4 hours at rest between TAP and LAI groups. **Figure S6.** Funnel plot of VAS score in 4 hours on movement between TAP and LAI groups. **Figure S7.** Funnel plot of VAS score in 24 hours at rest between TAP and LAI groups. **Figure S8.** Funnel plot of VAS score in 24 hours on movement between TAP and LAI groups. **Figure S9.** Funnel plot of mean morphine requirements (mg) 24 hours following surgery between TAP block and local infiltration. **Figure S10.** Funnel plot of postoperative nausea and vomiting (PONV) rate 24 hours following surgery between TAP block and local infiltration. (DOCX 350 KB)

## Abbreviations

LAI: Local anesthetic infiltration
PONV: Postoperative nausea and vomiting
RCT: Randomized controlled trial
TAP: Transversus abdominis plane
VAS: Visual analog scale.
