# Supplementary material for: Transversus abdominis-plane block versus local anesthetic wound infiltration in lower abdominal surgery: a systematic review and meta-analysis of randomized controlled trials
Source: BMC Anesthesiol. 2014 Dec 15;14:121. doi: 10.1186/1471-2253-14-121 (PMC4289539; doi:10.1186/1471-2253-14-121)
Supplement: Supplementary file 1 — Additional file 1: Figure S1: Risk of bias graph: review authors’ judgments about each risk of bias item presented as percentages across all included studies. Figure S2. Risk of bias summary: review authors’ judgments about each risk of bias item for each included study. Figure S3. Funnel plot of VAS score in 2 hours at rest between TAP and LAI groups. Figure S4. Funnel plot of VAS score in 2 hours on movement between TAP and LAI groups. Figure S5. Funnel plot of VAS score in 4 hours at rest between TAP and LAI groups. Figure S6. Funnel plot of VAS score in 4 hours on movement between TAP and LAI groups. Figure S7. Funnel plot of VAS score in 24 hours at rest between TAP and LAI groups. Figure S8. Funnel plot of VAS score in 24 hours on movement between TAP and LAI groups. Figure S9. Funnel plot of mean morphine requirements (mg) 24 hours following surgery between TAP block and local infiltration. Figure S10. Funnel plot of postoperative nausea and vomiting (PONV) rate 24 hours following surgery between TAP block and local infiltration. (DOCX 350 KB) [file 12871_2014_329_MOESM1_ESM.docx]

Figure 1. Risk of bias graph: review authors' judgments about each risk of bias item presented as percentages across all included studies.

Figure 2. Risk of bias summary: review authors' judgments about each risk of bias item for each included study.


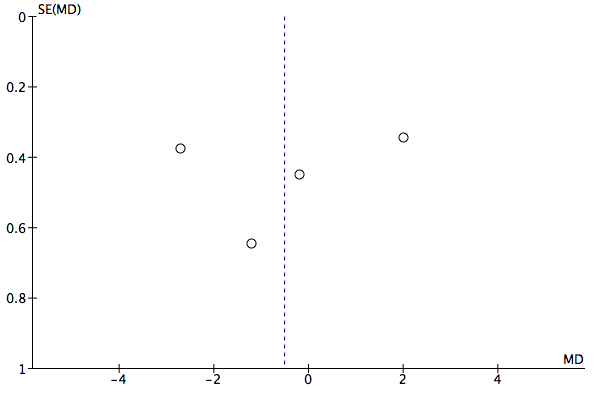


Figure 3. Funnel plot of VAS score in 2 hours at rest between TAP and LAI groups.


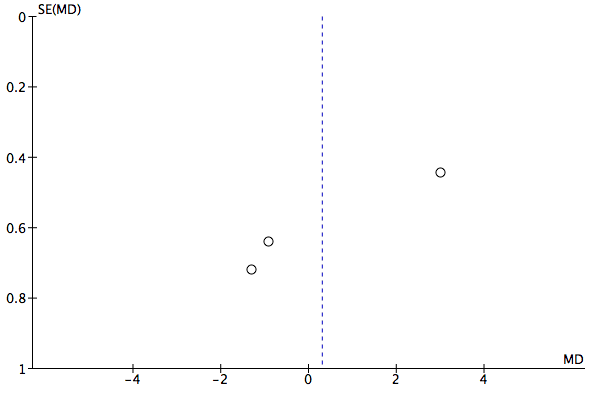


Figure 4. Funnel plot of VAS score in 2 hours on movement between TAP and LAI groups.


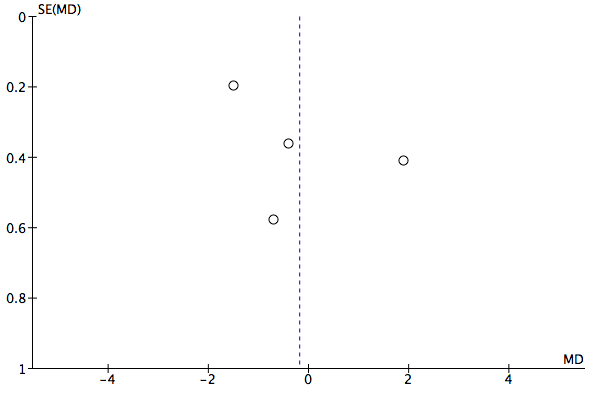


Figure 5. Funnel plot of VAS score in 4 hours at rest between TAP and LAI groups.


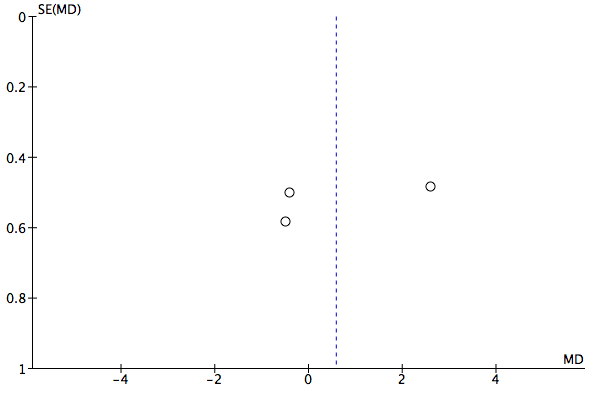


Figure 6. Funnel plot of VAS score in 4 hours on movement between TAP and LAI groups.


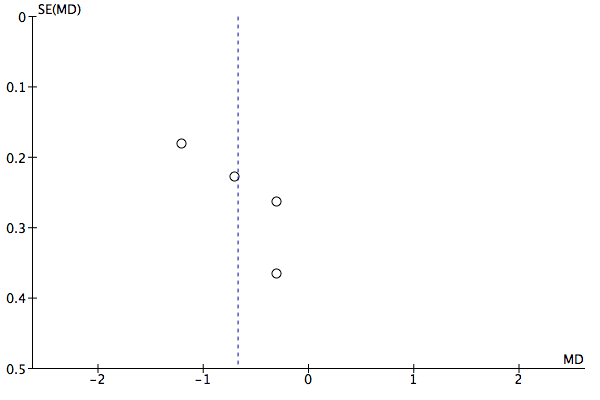


Figure 7. Funnel plot of VAS score in 24 hours at rest between TAP and LAI groups.


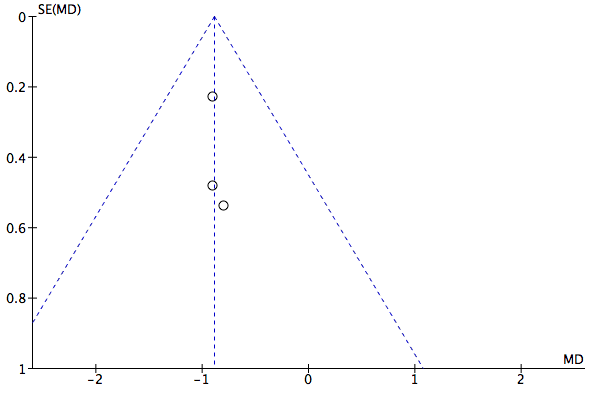


Figure 8. Funnel plot of VAS score in 24 hours on movement between TAP and LAI groups.


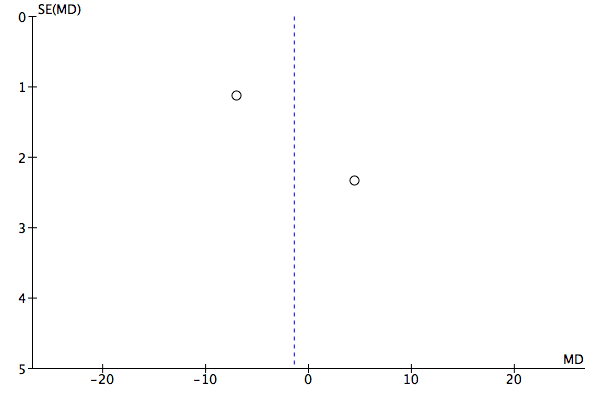


Figure 9. Funnel plot of mean morphine requirements (mg) 24 hours following surgery between TAP block and local infiltration.


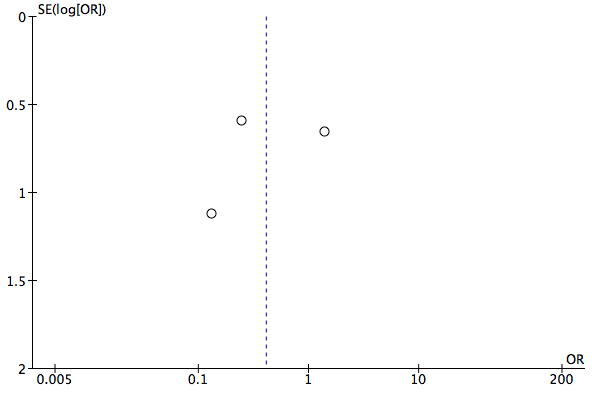


Figure 10. Funnel plot of postoperative nausea and vomiting (PONV) rate 24 hours following surgery between TAP block and local infiltration.
